# Clinicopathologic characteristics, pathology, and prognosis of 77 dogs with focal segmental glomerulosclerosis

**DOI:** 10.1111/jvim.15837

**Published:** 2020-07-07

**Authors:** Sarah K. Lorbach, Jessica A. Hokamp, Jessica M. Quimby, Rachel E. Cianciolo

**Affiliations:** ^1^ Department of Veterinary Clinical Sciences, College of Veterinary Medicine The Ohio State University Columbus Ohio USA; ^2^ Department of Veterinary Biosciences, College of Veterinary Medicine The Ohio State University Columbus Ohio USA; ^3^ International Veterinary Renal Pathology Service (IVRPS), Combined Service at The Ohio State University, Columbus, Ohio and Texas A&M College Station Texas USA

**Keywords:** global glomerulosclerosis, glomerular disease, nonimmune complex glomerulopathy, podocyte, protein losing nephropathy, proteinuria, renal biopsy

## Abstract

**Background:**

Focal segmental glomerulosclerosis (FSGS) is a common cause of nonimmune complex glomerulopathy and the prognosis and clinicopathologic findings associated with this condition have not been described in dogs.

**Objective:**

To characterize the presentation and identify clinical factors associated with the survival of dogs with FSGS.

**Animals:**

Seventy‐seven dogs diagnosed with FSGS based on evaluation of renal biopsy samples submitted to the International Veterinary Renal Pathology Service.

**Methods:**

Retrospective review of medical records of dogs biopsied for evaluation of proteinuria between January 2015 and May 2017.

**Results:**

The incidence of FSGS among all dogs biopsied for proteinuria was 26%. Significantly more females (48; 62.3%) than males (29; 37.7%) were affected (*P* = .04). At the time of biopsy, median serum creatinine concentration (SCr) was 1.2 mg/dL (range, 0.3‐8.7), median serum albumin concentration (Alb) was 2.8 g/dL (range, 1.1‐4.6), median systolic blood pressure was 153.5 mm Hg (range, 95‐260), and median urine protein : creatinine ratio was 5.9 (range, 1.4‐22). Median survival time after biopsy was 258 days (range, 26‐1003) for dogs that died from all causes (n = 32). Factors that were associated with a shorter survival time included SCr ≥ 2.1 mg/dL (*P* < .01) and Alb < 2 g/dL (*P* < .01).

**Conclusions and Clinical Importance:**

Most dogs with FSGS were female, and although commonly hypertensive, azotemia, severe hypoalbuminemia and ascites or edema were observed infrequently. Variables significantly associated with survival time were SCr and Alb.

**Abbreviations**
ACDs: all‐cause deaths
ACE: angiotensin converting enzyme
Alb: serum albumin concentration
ARB: angiotensin receptor blocker
CKD: chronic kidney disease
FPE: foot process effacement
FSGS: focal segmental glomerulosclerosis
GGS: global glomerulosclerosis
HT: hypertension
ICGN: immune‐complex glomerulonephritis
IVRPS: International Veterinary Renal Pathology Service
RRDs: renal‐related deaths
SBP: systolic blood pressure
SCr: serum creatinine concentration
UPC: urine protein : creatinine ratio
WSAVA: World Small Animal Veterinary Association

## 1 INTRODUCTION

Focal segmental glomerulosclerosis (FSGS) is the most common nonimmune complex glomerular disease in dogs with an incidence ranging from 20.6 to 33% of dogs that had comprehensive evaluation of renal tissue for clinical suspicion of proteinuric glomerular disease.^1^, ^2^ It is a leading cause of glomerular disease in humans with a prevalence of 22 to 38.9% in patients with nondiabetic primary glomerulopathy,^3^, ^4^ and often progresses to end‐stage renal disease.^5^ Importantly, FSGS describes a histopathologic pattern characterized by irreversible and progressive podocyte injury with accumulation of matrix (sclerosis) within glomerular tufts that compresses and eventually obliterates capillary lumens.^5^, ^6^, ^7^ In humans, FSGS is classified as primary (or idiopathic) or secondary (or acquired), the latter of which includes viral‐associated, drug‐associated, genetic, adaptive, and maladaptive forms.^5^, ^6^, ^8^ Glomerulosclerosis, whether primary or secondary, is a pathophysiologic response that occurs as a sequela to insult and subsequent loss of podocytes.^5^, ^9^ The podocyte, a terminally differentiated cell, exhibits an adaptive response to the loss of an adjacent podocyte or glomerular enlargement or both, wherein the podocyte stretches and hypertrophies, leading to podocyte foot process effacement (FPE), or alteration of the slit diaphragm.^5^, ^6^, ^7^, ^10^ If the insult persists, podocyte detachment occurs resulting in a denuded glomerular basement membrane. Adhesions between the denuded glomerular basement membrane and Bowman's capsule (synechiae) and synthesis of extracellular matrix within segments of the tuft develop. Segmentally sclerotic glomeruli typically progress to global glomerulosclerosis (GGS), but not all globally sclerotic glomeruli go through a stage of segmental sclerosis as evidenced in humans, in whom GGS occurs at an expected rate during normal aging.^5^, ^11^, ^12^, ^13^, ^14^

Although FSGS is well characterized in humans, little is known about its clinical presentation and progression in dogs. A genetically linked podocytopathy is suspected in some breeds. Mutations encoding proteins of the slit diaphragm have been identified in soft‐coated Wheaten Terriers and Airedale terriers leading to podocyte loss and development of FSGS.^15^, ^16^ The occurrence of FSGS also has been described in 8 related miniature schnauzers, but a mode of inheritance was not identified.^10^ The purpose of our study was to describe the clinical presentation of 77 dogs with FSGS, assess variables that might impact survival time, and provide clinical follow‐up data. We hypothesized that survival time would be negatively associated with the following variables: a serum creatinine concentration (SCr) ≥ 2.1 mg/dL, hypoalbuminemia (<2 g/dL), presence of ascites or edema, presence of systemic arterial hypertension (HT), a urine protein : creatinine ratio (UPC) ≥ 5.9 at the time of biopsy, and GGS affecting ≥25% of glomeruli (and therefore loss of >25% of nephron mass).

## 2 MATERIALS AND METHODS

### 2.1 Clinical data

The International Veterinary Renal Pathology Service (IVRPS) database was searched for dogs diagnosed with FSGS by renal biopsy sample evaluation between January 2015 and May 2017. The diagnosis of FSGS was based on evaluation of renal biopsy samples using standards set by the World Small Animal Veterinary Association (WSAVA) Renal Pathology Initiative.^7^ Briefly, evaluation of the sample included light microscopy (hematoxylin and eosin, periodic acid‐Schiff, Masson's trichrome, and Jones methenamine silver stains), immunofluorescence (IF), and transmission electron microscopy when available. Medical records were retrospectively reviewed for 77 dogs. Clinical data were acquired by reviewing submission forms that accompanied the biopsy sample, in addition to requesting additional history and follow‐up clinical data by phone from the submitting specialty care center or primary veterinarian. Signalment, date of biopsy, clinicopathologic data, presence of ascites or edema, medication history, urinalysis results, and cause of death were determined from review of the medical record. At the time of biopsy, clinicopathologic data included: SCr, serum albumin concentration (Alb), UPC, and systolic blood pressure (SBP). To maintain consistency with a previous study,^7^ presence of HT was defined as a recorded SBP repeatedly ≥160 mm Hg. Additionally, patients that were normotensive at the time of biopsy also were defined as HT if they had been hypertensive historically but were controlled on an antihypertensive medication (other than an angiotensin converting enzyme [ACE] inhibitor alone). In cases in which several pressures were measured in 1 session, the mean result was used. The presence of ascites or edema was determined by review of the medical record and diagnostic imaging reports close to the time of biopsy. The finalized IVRPS reports were used to calculate the percentage of GGS within the biopsy sample.

### 2.2 Outcome/survival post‐biopsy

A board‐certified veterinary internist with a special interest in nephrology (J. M. Q.) reviewed the records of all dogs that died (all‐cause deaths [ACDs]) to determine whether the cause of death could be determined and, if so, whether it was a renal‐related death (RRD). All‐cause death included any dog that died or was euthanized. Dogs classified as having RRD died or were euthanized because of clinical signs most likely associated with uremia or consequences of proteinuria (eg, thromboembolic disease) and had no other comorbidities thought to contribute to the cause of death or euthanasia. The end point for the study was ACD. Dogs were included in the survival data if they survived at least 1 week after biopsy and had a known date of death (including those that were euthanized). This excluded 3 dogs that did not have a known date of death, and an additional 3 dogs that died or were euthanized because of declining condition shortly after renal biopsy. The cause of death in these 3 latter dogs was as follows: noncardiogenic pulmonary edema and need for ventilatory support, a suspected thromboembolic event after a previously diagnosed portal vein thrombus and progressive clinical deterioration before hospital discharge. A survival of 1 week was selected to ensure all decisions regarding euthanasia were made in light of results from preliminary evaluation of the renal biopsy and to exclude any biopsy‐associated mortality. Survival was evaluated in association with the factors hypothesized to influence prognosis for both ACD and RRD subgroups (SCr, Alb, UPC at time of biopsy, highest UPC documented, HT, percent GGS, and presence of ascites or edema). For each variable, subgroups were determined based on specific definitions. A SCr of 2.1 mg/dL was used to divide patients into high and low azotemic groups, based on International Renal Interest Society (IRIS) staging guidelines before 2019.^17^ Systemic arterial HT at the time of biopsy or current use of an antihypertensive medication defined the subgroup with HT (as described above). An Alb of <2.0 g/dL was used to define severe hypoalbuminemia, which is consistent with studies evaluating protein‐losing enteropathies and the potential for increased risk of thrombosis.^18^, ^19^ For UPC, the lower quartile (3.9), median (5.9), and upper quartile (7.9) of the population all were utilized as different cut points. For GGS, the median percentage in dogs with RRD was approximately 25%, and therefore this percentage was used to categorize GGS into high and low subgroups.

### 2.3 Statistical analysis

Statistical analysis was performed using Stata version 16.0 (Stata Corp. LP, College Station, Texas). A 2‐sample test of proportion was used to test for a significant difference in sex distribution. The Shapiro‐Wilk test was used to assess normality of the residuals, and data were natural log or square root transformed as necessary. Descriptive statistics were presented as median (range). Simple linear regression was used to assess correlation between survival time and the continuous variables UPC (both at the time of biopsy and highest documented) and Alb. A Mann‐Whitney *U* test was used to test for a significant difference in survival time between dogs with ACD and RRD. Differences in survival time for factors hypothesized to influence prognosis (eg, presence or absence of HT, SCr < 2.1 mg/dL versus SCr ≥ 2.1 mg/dL; Alb < 2 g/dL versus Alb ≥ 2 g/dL; GGS < 25% versus GGS ≥ 25%; UPC at time of biopsy and highest documented UPC evaluated using each quartile as a cut point) were determined by Kaplan‐Meier survival analysis using the log‐rank test. Results were considered significant if *P* < .05.

## 3 RESULTS

### 3.1 Incidence and signalment

A total of 299 canine renal biopsy samples were submitted to the IVRPS for the clinical indication of proteinuria in the 28‐month period. Of those, 77 (26%) were diagnosed as FSGS. Dogs ranged in age from 2.3 to 14.8 years (median 9.5 years). There were significantly more females (48; 62.3%) than males (29; 37.7%; *P* = .04). Of the females, 47 were spayed and, of the males, 26 were castrated. In total, 31 different breeds were represented. The most common breeds included mixed breed dogs (n = 15, 19.5%), Labrador retrievers (n = 6, 7.8%), golden retrievers (n = 5, 6.5%), unspecified cocker spaniels (n = 5, 6.5%), and beagles (n = 4, 5.2%). Twenty‐six other breeds were represented with ≤3 dogs in each breed. Within this cohort, there were 3 soft‐coated Wheaten terriers, and 1 miniature schnauzer. Of the 15 mixed breed dogs, 10 were reported to be Yorkshire terrier, Maltese, cocker spaniel, or poodle mixes.

### 3.2 Pathology

Histologic findings were similar to those previously reported, and included segmental collapse (sclerosis) of the glomerular tuft, usually with associated hyalinosis (entrapped plasma proteins).^7^ Synechiae also were frequent. When frozen tissue containing glomeruli was available (57 dogs), IF staining was performed for immunoglobulin (Ig) G, IgM and IgA along with complement component C3, and lambda light chain to assess the presence of underlying immune complex deposition within the glomerulus. Weak and nonspecific IgM staining occasionally was identified within portions of the glomerulus exhibiting hyalinosis and sclerosis because plasma proteins were trapped in those regions, as has been reported in affected human patients.^5^ Transmission electron microscopy findings (available for 73 dogs) included FPE, a key feature consistent with podocyte injury,^6^ and further confirmed the absence of electron‐dense immune deposits within the glomerulus. Three dogs were diagnosed based on histology alone; none was included in the survival data. Diagnostic tests were not performed in these dogs for the following reasons: 2 dogs were euthanized within 3 days of being biopsied and their testing was canceled and the family of the other dog expressed financial concerns. Within renal biopsy samples, the median number of glomeruli available for interpretation was 17 (range, 4‐60). The median (range) percentage of GGS in all 77 dogs, and in subpopulations of ACD, RRD and dogs alive at the time of follow‐up are presented in Table 1.

**TABLE 1:** Clinicopathologic and histologic characteristics of 77 dogs with focal segmental glomerulosclerosis at the time of biopsy

	All dogs biopsied	All‐cause deaths	Renal‐related deaths	Alive at data collection
	n = 77	n = 38	n = 18	n = 23
Serum creatinine (mg/dL)	1.2 (0.3‐8.7)	1.45 (0.5‐8.7)	1.93 (0.5‐8.7)	0.9 (0.3‐2.09)
	n = 75	n = 38	n = 18	n = 23
Serum albumin (g/dL)	2.8 (1.1‐4.6)	2.6 (1.1‐4.6)	2.2 (1.1‐3.5)	3 (1.3‐4)
	n = 7 3	n = 38	n = 18	n = 22
Systolic blood pressure (mm Hg)	153.5 (95‐260)	158.5 (110‐250)	160 (110‐250)	147.5 (95‐260)
	n = 70	n = 34	n = 15	n = 22
UPC	5.9 (1.4‐22)	5.9 (1.4‐14.8)	4.7 (1.4‐13.4)	5.5 (1.86‐22)
	n = 74	n = 37	n = 17	n = 22
Global glomerulosclerosis (%)	14 (0‐67)	17 (0‐67)	23.5 (0‐67)	13 (0‐44)
	n = 77	n = 38	n = 18	n = 23

*Note:* Median (range).

Abbreviation: UPC, urine protein : creatinine ratio.

### 3.3 Clinicopathologic data

Median and range of selected clinicopathologic data at the time of biopsy are presented in Table 1. Sixty‐one of 73 (84%) dogs had Alb ≥ 2 g/dL. Documentation of SBP at the time of or before biopsy was available in 72 dogs, and HT was present in 44 (61%) dogs. Although all 77 dogs were reportedly proteinuric before biopsy, UPCs were only available in 74 dogs at the time of biopsy. The median duration of proteinuria before biopsy (reported in 44 dogs) was 108.5 days (range, 5‐1368 days). No correlation was found between magnitude of UPC (either at time of biopsy or highest documented) and Alb in this cohort of dogs. The presence or absence of ascites or edema was known in 45 dogs and ascites or edema was present in 6 (13%). Three dogs had ascites, 2 had peripheral edema, and 1 dog had both. For the 6 dogs with ascites or edema, the median Alb at the time of biopsy was 1.5 g/dL (range, 1.1‐2.1 g/dL) and the median UPC was 5.9 (range, 2.1‐22). The median SCr of these 6 dogs was 1.45 mg/dL (range, 0.6‐8.7 mg/dL). Systolic blood pressure was known in 4 of the dogs with ascites or edema and all had HT (median, 167 mm Hg; range, 160‐180 mm Hg). Three of the dogs with ascites or edema were diagnosed with nephrotic syndrome: specifically, 1 was reported to be nephrotic on the submission form and the other 2 dogs had high serum cholesterol concentrations consistent with the diagnosis (343 mg/dL and 359 mg/dL, respectively).

### 3.4 Survival post‐biopsy

**FIGURE 1:**
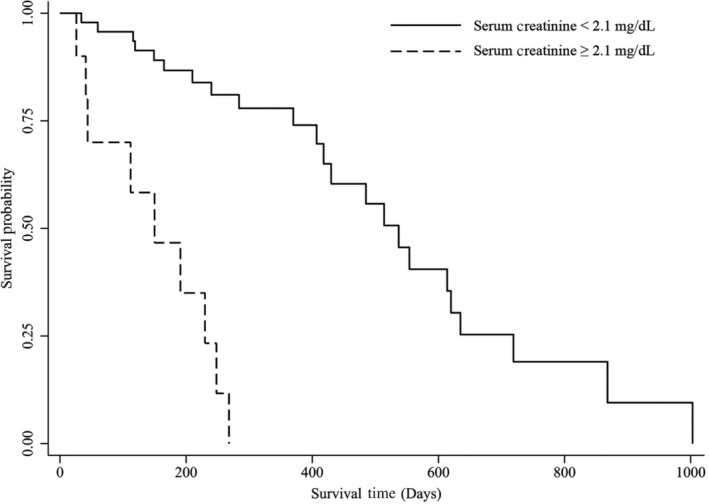
Kaplan‐Meier survival curve for serum creatinine in dogs with focal segmental glomerulosclerosis dying from all‐cause death. Median survival time post‐biopsy in dogs dying from all‐cause death was significantly shorter in dogs with serum creatinine ≥ 2.1 mg/dL (*P* < .001)

**FIGURE 2:**
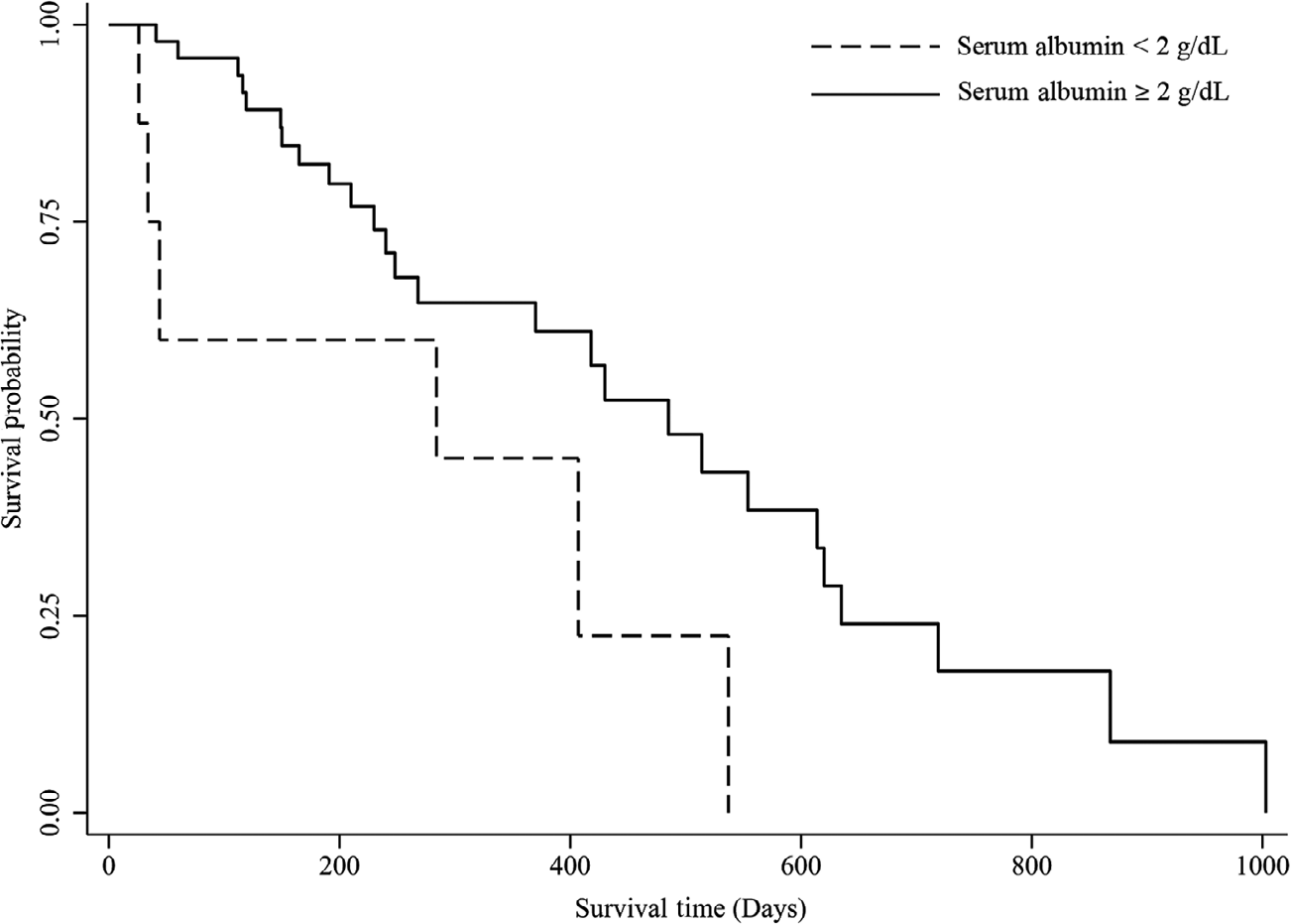
Kaplan‐Meier survival curve for serum albumin in dogs with focal segmental glomerulosclerosis dying from all‐cause death. Median survival time post‐biopsy in dogs dying from all‐cause death was significantly shorter in dogs with serum albumin < 2.0 g/dL (*P* = .03)

At the time of data collection, 23 dogs (30%) were alive, 38 (49%) were dead, and 16 (21%) were lost to follow‐up. Of the 38 ACD dogs, 18 (47%) were determined to be RRD based on medical record review. When only dogs surviving >1 week post‐biopsy and with a defined date of death were included in survival analysis, 32 ACD dogs and 15 RRD dogs remained. Median survival time post‐biopsy was 258 days (range, 26‐1003 days) for ACD dogs and 268 days (range, 41‐635 days) for RRD dogs, which was not significantly different. Survival times (ranges) for hypothesized prognostic variables are presented in Table 2. The only variables significantly associated with survival time were SCr for both RRD and ACD dogs (*P* < .001), and Alb concentration for ACD dogs (*P* = .03; Figures 1 and 2). No correlation was found between either UPC at time of biopsy or highest UPC documented and survival time, regardless of the cut point used. Three of the 6 dogs with ascites or edema did not survive >1 week and could not be included in the survival data. Two dogs with ascites or edema were still alive at the time of data collection and the only dog included in survival analysis lived 407 days post‐biopsy. Median duration of time until follow‐up for dogs alive at the time of data collection was 240 days (range, 39‐800 days). Prognostic variables for the dogs still alive at data collection are presented in Table 2.

**TABLE 2:** Association of survival time post‐biopsy (days) with clinical, clinicopathologic, and histologic categories at the time of diagnosis in 77 dogs diagnosed with focal segmental glomerulosclerosis

	All‐cause deaths (n = 32)			Renal‐related deaths (n = 15)			Alive at data collection (n = 23)
	No. of dogs	Median (range)	*P* value	No. of dogs	Median (range)	*P* value	No. of dogs
Serum creatinine							
<2.1 mg/dL	23	418 (34‐1003)	**<.001**	8	466 (116‐635)	**<.001**	23
≥2.1 mg/dL	9	150 (26‐268)		7	150 (41‐268)		0
Serum albumin							
<2 g/dL	6	164 (26‐537)	**.03**	3	284 (44‐407)	.19	2
≥2 g/dL	26	258 (41‐1003)		12	258 (41‐635)		20
Systemic arterial hypertension							
No	10	413 (41‐1003)	.07	5	407 (41‐514)	.49	11
Yes	21	230 (26‐719)		9	248 (44‐635)		11
UPC							
<5.9	14	239 (41‐1003)	.75	8	258 (41‐635)	.52	12
≥5.9	18	389 (26‐868)		7	407 (44‐554)		10
Global glomerulosclerosis							
<25%	22	262 (26‐1003)	.58	8	238 (44‐620)	.1	19
≥25%	10	258 (34‐868)		7	268 (41‐635)		4
Ascites or edema							
No	28	327 (26‐1003)	.99	8	266 (112‐635)	.53	10
Yes	1	407		1	407		2

*Note:* Kaplan‐Meier survival analysis with log‐rank test was used to determine significant differences between prognostic factor categories. Bolded *p* values are statistically significant.

Abbreviation: UPC, urine protein : creatinine ratio.

### 3.5 Treatment

Treatments prescribed were inconsistently available in the medical record review. An ACE inhibitor was prescribed in 60/61 dogs (98%), and angiotensin receptor blockers (ARBs) were used in 27/47 dogs (57%) for which data were available. The only dog not known to be on an ACE inhibitor was being treated with an ARB. Twenty‐six dogs were treated with both an ACE inhibitor and ARB simultaneously. A renal diet was prescribed in 39/48 dogs (81%), amlodipine was prescribed in 30/46 dogs (65%), antithrombotic treatment was used in 37/47 dogs (79%), and omega‐3 fatty acid supplementation was used in 24/41 dogs (59%) for which data were available.

Based on available data within medical records, 5 dogs had been treated with immunosuppressive drugs before biopsy; mycophenolate (2 dogs), prednisone (1 dog), cyclosporine (1 dog), or azathioprine (1 dog). The dog treated with prednisone had been diagnosed with immune‐mediated hemolytic anemia 6 months before and was tapered off prednisone 7 weeks before biopsy although its UPC remained high. Mycophenolate was administered 1 and 3 months before biopsy in 2 different dogs for a duration of 1 month each. Azathioprine was administered in 1 dog starting 3 months before biopsy and discontinued after the biopsy results were available. Cyclosporine was administered 4 months before biopsy in 1 dog and was discontinued when the UPC and SCr increased while on treatment. While on immunosuppressive therapy, all dogs experienced lack of improvement or some increase in their UPC, although none of the dogs decompensated clinically as a result of empirical immunosuppressive treatment.

## 4 DISCUSSION

Focal segmental glomerulosclerosis is a common cause of glomerular disease in dogs biopsied for evaluation of proteinuria. Clinicopathological variables significantly associated with survival time in dogs diagnosed with FSGS were SCr and Alb. A significantly shorter survival time after biopsy was documented in ACD and RRD dogs with SCr ≥ 2.1 mg/dL and in ACD dogs that were severely hypoalbuminemic (Alb < 2.0 g/dL). Interestingly, UPC at biopsy, highest documented UPC, and Alb were not associated with survival time. In this cohort, the majority of dogs were female, neither azotemic nor severely hypoalbuminemic, but were commonly hypertensive and infrequently found to have ascites or edema.

Proteinuria has been associated with increased risk of uremic crisis along with progression of chronic kidney disease (CKD) in veterinary and human patients.^20^, ^21^, ^22^, ^23^, ^24^ Dogs with CKD and UPC ≥ 3 have been shown to have higher relative risk of uremic crisis than those with UPC ≤ 1.^20^ In our cohort of dogs, median UPC at biopsy was 5.9. Therefore, we hypothesized that proteinuria would be a factor associated with disease progression and shorter survival time. However, the magnitude of proteinuria at the time of biopsy was not found to be associated with survival in our cohort of dogs. Although clinical factors such as medical treatment for proteinuria and systemic arterial HT and the timing of the renal biopsy in the disease course could impact the magnitude of UPC at the time of biopsy,^25^ the highest documented UPC also was not found to correlate with survival time. It is plausible that UPC may not be the best biomarker of glomerular scarring. Our previous studies have shown that the magnitude of urinary IgM and IgG in dogs correlated better with glomerular damage observed in biopsy samples than did UPC. Urinary IgM and IgG also were significantly associated with shorter survival time post‐biopsy whereas UPC was not,^26^ indicating that specific urinary proteins, as opposed to overall magnitude of proteinuria, may be more prognostic. Similarly in humans, excretion of IgG and IgM better predicts progression to CKD as opposed to quantification of proteinuria over 24 hours.^27^ Additionally, the timing of biopsy might have an effect on any association between UPC and survival post‐biopsy. Proteinuria can occur variably in the progression of glomerular disease, but appears to occur most commonly after FPE.^9^, ^28^ The time at which dogs in this cohort were biopsied in relation to development of proteinuria was highly variable and the stage of their disease when proteinuria was first identified was unknown, which could impact ability to predict survival.

Magnitude of proteinuria cannot definitively differentiate immune‐complex glomerulonephritis (ICGN) and non‐ICGN. In a previous European study of 162 dogs with proteinuria, UPC >12.5 was suggested as a cutoff because no dog with non‐ICGN had a higher UPC.^1^ Although their classification of ICGN and non‐ICGN was slightly different from that of our cohort, review of the results from that study showed that dogs with ICGN had higher UPC than those diagnosed with FSGS. However, 7 (9%) dogs in our cohort had UPC > 12.5 and the UPC of dogs with FSGS in a WSAVA renal pathology study ranged from 2.6 to 24.5,^7^ indicating that dogs with FSGS can be severely proteinuric, and this cutoff cannot definitively differentiate ICGN from non‐ICGN cases.

Despite substantial proteinuria affecting dogs in our study, few had severe hypoalbuminemia. However, when present, severe hypoalbuminemia was associated with shorter survival time in ACD dogs. These findings are similar to those of a previous study in dogs with CKD in which hypoalbuminemia was found to be associated with shorter survival.^29^ In the European study of 162 dogs with renal biopsies, those with FSGS were less likely to have hypoalbuminemia than those with ICGN (specifically, membranoproliferative and mixed glomerulonephritis).^1^ The cohort of dogs with FSGS in the WSAVA renal pathology study had a median Alb of 2.5 g/dL (range, 1.3‐4.1 g/dL).^7^ Additionally, in the study of 8 related miniature schnauzers with FSGS, 5 dogs were found to have normal Alb with only mild to moderate hypoalbuminemia (2.3‐2.5 g/dL) in the other 3 dogs.^10^ Although we did not make comparisons between patients with FSGS and the IVRPS cases diagnosed with ICGN over the same time period, collectively these data support the finding that dogs with FSGS largely maintain normal to mildly decreased Alb.

In our study, a significant correlation between Alb and UPC was not found, which confounds the prior assumption that severe proteinuria alone is responsible for hypoalbuminemia in dogs with glomerular disease.^1^ Similar findings have been reported in humans with secondary adaptive FSGS where normal Alb was present despite nephrotic‐range proteinuria (ie, 24‐hour proteinuria of ≥3.5 g/d).^6^, ^30^ It is hypothesized that potentially slow onset of proteinuria in patients with normal Alb may allow systemic compensation.^30^ Although dogs in our cohort were diagnosed with proteinuria a median of approximately 3.5 months before biopsy, the rapidity of onset of their proteinuria is unknown. It is also important to consider that Alb may be lower in dogs with other types of glomerular disease for reasons beyond urinary losses, such as in cases of inflammation‐associated acute phase reactions.^31^ Qualitative analysis of urinary proteins and biomarkers in dogs with FSGS may advance our understanding of proteinuria and its relationship to Alb in these patients.

In people, primary FSGS is treated using immunosuppressive drugs, plasmapheresis, and immunoadsorption.^32^ Secondary FSGS, specifically adaptive and genetic forms, most commonly is managed initially with antiproteinuric treatment and often is associated with more mild clinical presentation as compared to primary FSGS.^5^, ^33^ Although it was not an aim of our study, we documented that dogs in our cohort treated with immunosuppressive drugs before renal biopsy did not exhibit improvement in their UPC results. Classification into primary or secondary FSGS is a complex process in people, and was not performed in the dogs in our study.^6^

In our cohort, dogs with ascites or edema were not found to have a significantly shorter survival time compared to those that did not. However, 3 of the 6 dogs with ascites or edema were dead within 1 week, and therefore were not included in the survival statistics. Therefore, presumably the presence of ascites or edema does affect survival. Despite this, the overall occurrence of ascites or edema in our cohort of dogs was relatively low (13%). Findings were similar in the European study of 162 dogs with proteinuria where only 1 (9.1%) of 11 dogs with FSGS and a known body fluid status had ascites. In comparison, of 40 dogs with ICGN and known body fluid status in that study, 15 (37.5%) had ascites.^1^ Of the 6 dogs in our cohort with ascites or edema, 1 was reported to be nephrotic and 2 dogs had hypercholesterolemia and hypoalbuminemia consistent with nephrotic syndrome. Last, none of 8 related miniature schnauzer dogs with FSGS were described to have ascites or edema.^10^ Although few dogs with FSGS appear to develop ascites or edema, this finding may be confounded if clinicians are less inclined to biopsy dogs with this clinical finding.

Systemic arterial HT either before or at the time of biopsy was a common finding in our cohort of patients, affecting 61% of dogs. Similarly, 14 of 26 dogs (53.8%) with FSGS in the WSAVA renal pathology cohort were found to have HT,^7^ and 4 of 8 miniature schnauzer dogs with FSGS were known to have HT.^10^ In dogs with CKD, HT is associated with increased risk of uremic crisis and shorter survival times.^34^, ^35^, ^36^ Although CKD and HT have been evaluated in humans and dogs, few studies have evaluated SBP and glomerular disease. In a study of 443 adults and children with primary proteinuric glomerulopathies, 147 were diagnosed with FSGS, and of those, 65% were HT at the baseline of their data collection.^37^ The same study, when evaluating the adults with glomerulopathies, found that those with HT were at higher risk of developing end‐stage renal disease in addition to a decrease of 40% or more in their estimated glomerular filtration rate.^37^ Proteinuria, a characteristic of all dogs in our study, also is considered to be a clinical sign of target organ damage resulting from the effects of increased SBP on the kidney.^38^ In a study of 45 dogs, those with a UPC ≥1 were found to have a mean SBP of 164 ± 23 mm Hg, which was significantly higher than those with UPC < 1.0 (139 ± 18 mm Hg).^20^ Conclusions or statistical comparisons between SBP and UPC could not be accurately performed in our cohort of dogs because the method of measurement for SBP was not standardized among dogs in this cohort. Given that all dogs in our cohort had UPC ≥ 1.4, the percentage of dogs that had HT at the time of or before biopsy was not surprising.

In our cohort of dogs, the percentage of GGS was not found to be associated with survival time. Focal segmental glomerulosclerosis is known to progress to GGS, and in humans, the percentage of GGS has been associated with decreased glomerular filtration rate and progression to end‐stage renal disease.^5^, ^13^, ^14^, ^39^ In experimental rodent models, FSGS and GGS develop as podocyte loss surpasses 20% and 60% depletion, respectively, and podocyte depletion has been associated with severity of proteinuria.^28^, ^40^ The lack of correlation between percentage of GGS and survival time may be a consequence of the wide range in the timing of the renal biopsy as compared to the course of the patients' disease process.^6^ The duration of proteinuria before renal biopsy ranged from 5 to 1368 days, which allows for substantial variation in the development of sclerotic lesions. Additionally, the biopsy sample core might not be representative of the actual proportion of GGS in the kidney. Progression of FSGS to GGS also could not be evaluated because no dogs in the cohort underwent serial renal biopsies or had necropsy samples submitted to the IVRPS.

Limitations of our study are largely related to the retrospective nature of data collection. To ensure the ability to acquire records from primary and referring veterinarians, no biopsies before 2015 were included in the study. Although we report data from the largest cohort of dogs with a definitive diagnosis of FSGS based on comprehensive evaluation of renal tissue, the population size still is relatively small. Consistent with guidelines recommending standard treatment in dogs with glomerular disease,^38^ all patients with a known medication history were treated with an ACE inhibitor or ARB with or without other concurrent medications. This is likely a confounding factor in the assessment of proteinuria, HT, and prognosis because the study population was not controlled for dosage or frequency of these medications, and it was challenging to determine the extent to which adequate control was achieved. Additionally, it was not possible to obtain detailed dietary information, specifically the amount of protein in the diet, which has been demonstrated to improve survival in dogs with renal disease.^41^ Methods used to obtain blood pressure measurements were not standardized and pressures were obtained using both oscillometric and Doppler devices. Similarly, the collection method for urine samples was not standardized and samples may have been collected at home or in the hospital, with some representing results from pooled collections and some not.^42^ An additional limitation was difficulty in retrospectively differentiating between renal and nonrenal causes of death, especially in patients with comorbidities. As with all veterinary studies evaluating survival, the impact of euthanasia must be considered in interpretation of results.

In conclusion, we described clinicopathologic and histopathologic characteristics of 77 dogs with FSGS. In our cohort of 77 dogs, most were female, and although commonly hypertensive, were infrequently azotemic, severely hypoalbuminemic, or found to have ascites or edema. Median survival time was 258 days (range, 26‐1003 days) in this cohort of FSGS patients. An important limitation however was inability to determine how adequately HT and proteinuria were controlled, which could have influenced outcome. Additional veterinary studies are needed to further characterize FSGS, to obtain more information about this disease in dogs, and to prospectively evaluate survival in relation to treatment of proteinuria and HT.

## CONFLICT OF INTEREST DECLARATION

Authors declare no conflict of interest.

## OFF‐LABEL ANTIMICROBIAL DECLARATION

Authors declare no off‐label use of antimicrobials.

## INSTITUTIONAL ANIMAL CARE AND USE COMMITTEE (IACUC) OR OTHER APPROVAL DECLARATION

Authors declare no IACUC or other approval was needed.

## HUMAN ETHICS APPROVAL DECLARATION

Authors declare human ethics approval was not needed for this study.
